# Influence of type I IFN signaling on anti-MOG antibody-mediated demyelination

**DOI:** 10.1186/s12974-017-0899-1

**Published:** 2017-06-24

**Authors:** Carsten Tue Berg, Reza Khorooshi, Nasrin Asgari, Trevor Owens

**Affiliations:** 1 Institute of Molecular Medicine, Neurobiology, University of Southern Denmark, JB. Winsloewsvej 25, 5000 Odense C, Denmark; 2 Department of Neurology, Slagelse Hospital, Institute of Regional Health Service Research, University of Southern Denmark, Odense, Denmark

**Keywords:** Beta-interferon, Demyelination, Histopathology, Immunology, Animal model, Experimental autoimmune encephalomyelitis, Multiple sclerosis

## Abstract

**Background:**

Antibodies with specificity for myelin oligodendrocyte glycoprotein (MOG) are implicated in multiple sclerosis and related diseases. The pathogenic importance of anti-MOG antibody in primary demyelinating pathology remains poorly characterized.

**Objective:**

The objective of this study is to investigate whether administration of anti-MOG antibody would be sufficient for demyelination and to determine if type I interferon (IFN) signaling plays a similar role in anti-MOG antibody-mediated pathology, as has been shown for neuromyelitis optica-like pathology.

**Methods:**

Purified IgG2a monoclonal anti-MOG antibody and mouse complement were stereotactically injected into the corpus callosum of wild-type and type I IFN receptor deficient mice (IFNAR1-KO) with and without pre-established experimental autoimmune encephalomyelitis (EAE).

**Results:**

Anti-MOG induced complement-dependent demyelination in the corpus callosum of wild-type mice and did not occur in mice that received control IgG2a. Deposition of activated complement coincided with demyelination, and this was significantly reduced in IFNAR1-KO mice. Co-injection of anti-MOG and complement at onset of symptoms of EAE induced similar levels of callosal demyelination in wild-type and IFNAR1-KO mice.

**Conclusions:**

Anti-MOG antibody and complement was sufficient to induce callosal demyelination, and pathology was dependent on type I IFN. Induction of EAE in IFNAR1-KO mice overcame the dependence on type I IFN for anti-MOG and complement-mediated demyelination.

**Electronic supplementary material:**

The online version of this article (doi:10.1186/s12974-017-0899-1) contains supplementary material, which is available to authorized users.

## Introduction

Multiple Sclerosis (MS) is an inflammatory demyelinating disease of the central nervous system (CNS) with a complex pathogenesis [1]. MS pathology has been characterized by chronic inflammation, which leads to focal plaques of demyelination in the white matter [2]. More than 50% of MS lesions show complement (C) and immunoglobulin G (IgG) deposition on demyelinating axons, classified as type II pathology [3, 4], but there is no consensus as to either the specificity or pathologic role of IgG antibodies in blood or cerebrospinal fluid (CSF) of MS patients [1]. Plasma exchange, which removes IgGs, as well as immune complexes and cytokines, was effective for a group of patients confirmed to have had type II MS pathology [5]. This suggests that antibody and C are effectors of pathology in MS. Localization of C deposition has been shown in areas of active demyelination in patients with IgG myelin oligodendrocyte glycoprotein (MOG)-antibody-associated encephalomyelitis and pattern II MS [6–8]. One candidate autoantigen in MS is MOG, which is expressed by oligodendrocytes and on the outermost surface of the myelin sheath [9, 10]. Autoantibodies against MOG are implicated in pediatric MS [11] and acute disseminated encephalomyelitis (ADEM) [12] as well as in a subset of water channel aquaporin-4 (AQP4)-IgG seronegative neuromyelitis optica spectrum disorder (NMOSD) [13–15].

The most extensively used animal model of MS, experimental autoimmune encephalomyelitis (EAE), is an adjuvant-driven experimental autoimmune disease, which in most formulations is dependent on CD4+ T cells. In a commonly used EAE variant, immunization of C57BL/6 mice with an encephalitogenic peptide (MOG p35–55) induces a chronic inflammatory demyelinating disease that has been shown to be independent of IgG or B cells [16], whereas immunization with recombinant human MOG or a fusion protein of myelin basic protein and proteolipid protein induces EAE that is dependent on B cells and antibody [17–20]. It has previously been shown that injection of monoclonal antibody against MOG into Lewis rats with EAE induced demyelination [21, 22]. Serum from guinea pigs with chronic-relapsing EAE could also induce demyelination, when transferred into subarachnoid space of normal rats and the degree of demyelination correlated with high titers of anti-MOG antibody in serum [22]. However, direct demonstration of demyelination induced by MOG-specific antibody has not been reported. We have previously demonstrated complement-dependent astrocytopathology and demyelination in mice that received neuromyelitis optica (NMO) patient-derived IgG [23]. We also showed a requirement for type I IFN signaling for NMO-like pathology, showing that it was reduced in mice lacking the receptor for type I IFN [24]. This observation aligned with clinical findings that recombinant IFN-β is not an effective therapy for NMO whereas it shows effect for MS [25–27]. Interestingly, MOG-IgG-positive patients also showed increased disease activity under treatment with IFN-β [14, 28]. The objective of this study was to extend those findings, to investigate whether IFNAR dependency would be seen for other antibody specificities. We chose to test a monoclonal MOG-specific antibody. This required establishing that anti-MOG antibody is sufficient to induce primary demyelinating pathology and then to ask whether and how type I IFN signaling affects that pathology in mice with and without EAE.

## Materials and methods

### Mice

Adult female C57BL/6 mice aged 8–12 weeks were purchased from Taconic Europe (Ry, Denmark), and adult female type I IFN receptor deficient mice (IFNAR1-KO) aged 8–12 on C57BL/6 background were bred from mice originally provided by Dr. Marco Prinz. NOD-Scid/J immunodeficient mice aged 8–12 with impaired T and B cell lymphocyte development were obtained from Prof. Moustapha Kassem. All experiments were conducted in accordance with the Danish Animal Experiments Inspectorate (approval number 2014-15-0201-00369).

### EAE induction

C57BL/6 and IFNAR1-KO mice were immunized with MOG p35–55 (sequence MEVGWYRSPFSRVVHLYRNGK), obtained from TAG Copenhagen A/S in Denmark. Emulsions of MOG p35–55 (300 μg) and complete Freund’s adjuvant with heat-inactivated *Mycobacterium tuberculosis* (200 μg; BD-Biosciences, Sparks, USA) were injected subcutaneously into each hind flank. Animals received an intraperitoneal injection of pertussis toxin (0.3 μg; Sigma-Aldrich, Brøndby, Denmark) at the time of immunization and 2 days post-immunization. Mice were monitored for the loss of body weight and EAE symptoms as described previously [29].

### Intracorpus callosum injection

Mice were anesthetized using hypnorm (fluanisone/fentanyl) in combination with midazolam. Intra-corpus callosum (CC) injections were performed using the following stereotactic coordinates relative to bregma: 1 mm anterior, 1 mm lateral, and −1.6 ventral. Anti-MOG IgG2a (15 μg/mouse) (protein G affinity purified supernatant from hybridoma clone Z2 developed by Prof. Chris Linington and IgG2a isotype control from murine myeloma (Sigma-Aldrich, Brøndby, Denmark) were used in the experiments. Mice received intra-CC injection (5 μl) of anti-MOG Ab or control IgG2a together with 2 μl mouse C (Sigma-Aldrich; Cedarlane, Burlington, Canada). The mice were euthanized 2 days post-injection with an overdose of pentobarbital (0.2 mg per gram body weight, Glostrup Apotek, Glostrup, Denmark) and perfused transcardially with ice-cold phosphate buffered saline (PBS). For q-RT-PCR analysis, CNS tissue were dissected and immediately placed in TRIzol reagent and stored at −80 °C until further q-RT-PCR processing. For histology, mice were additionally perfused with 4% PFA in PBS. After removal, CNS tissue was post-fixed in 4% paraformaldehyde, immersed in 30% sucrose in PBS at 4 °C overnight, frozen with liquid nitrogen and stored at −80 °C until sections were cut on a cryostat.

### RNA extraction, reverse transcription, and quantitative real-time PCR

RNA was extracted from brain tissue using TRIzol reagent (Invitrogen-Molecular Probes, Eugene OR USA) in accordance with the manufacture’s protocol. One microgram of total RNA was reverse transcribed using M-MLV reverse transcriptase (Invitrogen) according to the manufacturer’s protocol. The following sequence-specific primers and probes were used for IFN-β and CXCL10: IFN-β: *For*: GCGTTCCTGCTGTGCTTCTC; *Rev*: TTGAAGTCCGCCCTGTAGGT; *Probe*: CGGAAATGTCAGGAGCT; CXCL10: *For*: GCCGT CATTTTCTGCCTCAT; *Rev*: GGCCCGTCATCGATATGG; *Probe*: GGACTCAAGGGATCC. All samples were run as triplicates and plates detecting the same gene were normalized using a generalized sample of cDNA. The relative quantitation of gene expression was determined using the delta cycle threshold (ΔCt) method.

### Histochemistry and immunohistochemistry

For routine histology, 12-μm thick sections were cut from frozen brain and spinal cord tissue and mounted on Superfrost® Plus (Thermo Scientific, Braunschweig, Germany) slides and stained using luxol fast blue (LFB) and hematoxylin and eosin (H&E) staining. Immunohistochemical staining used to identify and examine C deposition, astrocyte and microglia activation were performed by sequential antibody incubations and detection. The primary antibody used to identify C deposition, activation of astrocytes, and microglia were C9neo (rabbit polyclonal anti-C5b-9, 1:100, Abcam, Cambridge, UK), GFAP (rabbit anti-mouse glial fibrillary acidic protein, 1:500, Dako, Glostrup, Denmark), and Iba-1 (rabbit anti-ionized calcium binding adapter molecule 1 (1:500, Waco, Osaka, Japan), respectively. The secondary antibodies were goat anti-rabbit conjugated with biotin (Abcam, 1:1, Cambridge, UK) followed by streptavidin-horseradish peroxidase (streptavidin-HRP, 1:200, Amersham Biosciences, Little Buckinghamshire, UK) and DAB detection (3,3′-diaminobenzidine, 0.5 mg/ml, Sigma Aldrich). For double staining for infiltration of CD45-positive cells and laminin, frozen brain sections were incubated with primary antibodies––rat anti-mouse CD45 conjugated with PE (BD Biosciences, San Diego, CA, USA (1:300)) and rabbit anti-mouse laminin (Cederlane 1:100)––and the corresponding fluorophore-conjugated secondary antibody––donkey anti-rabbit IgG conjugated with Alexa fluor 488 (Invitrogen 1:200). Cellular nuclei were visualized by the use of 4,6-diamino-2-phenylindol (DAPI, Invitrogen) diluted in PBS.

### Quantitation of histological and immunohistochemical findings

For all analyses, brain and spinal cord sections were coded, analyzed, and quantified blindly. The area of demyelination in CC was quantitated using ImageJ software. The number of infiltrating cells in the CC was determined manually by counting the number of CD45 positive cells. The evaluation of the activation of astrocytes and microglia in and around demyelinated areas was performed blinded and semi*-*quantitatively using a scale from 0 to 3 as described [23].

### Data presentation and statistical analysis

All experiments were repeated at least three times, and data are presented as mean ± standard error of mean (SEM). Statistical significance was assessed by the two-tailed Mann–Whitney *U* test using GraphPad prism v. 6.0e software (GraphPad Software, USA). *P* values <0.05 were considered as evidence of statistical significance.

## Results

### Callosal demyelination by anti-MOG and complement

**Fig. 1:**
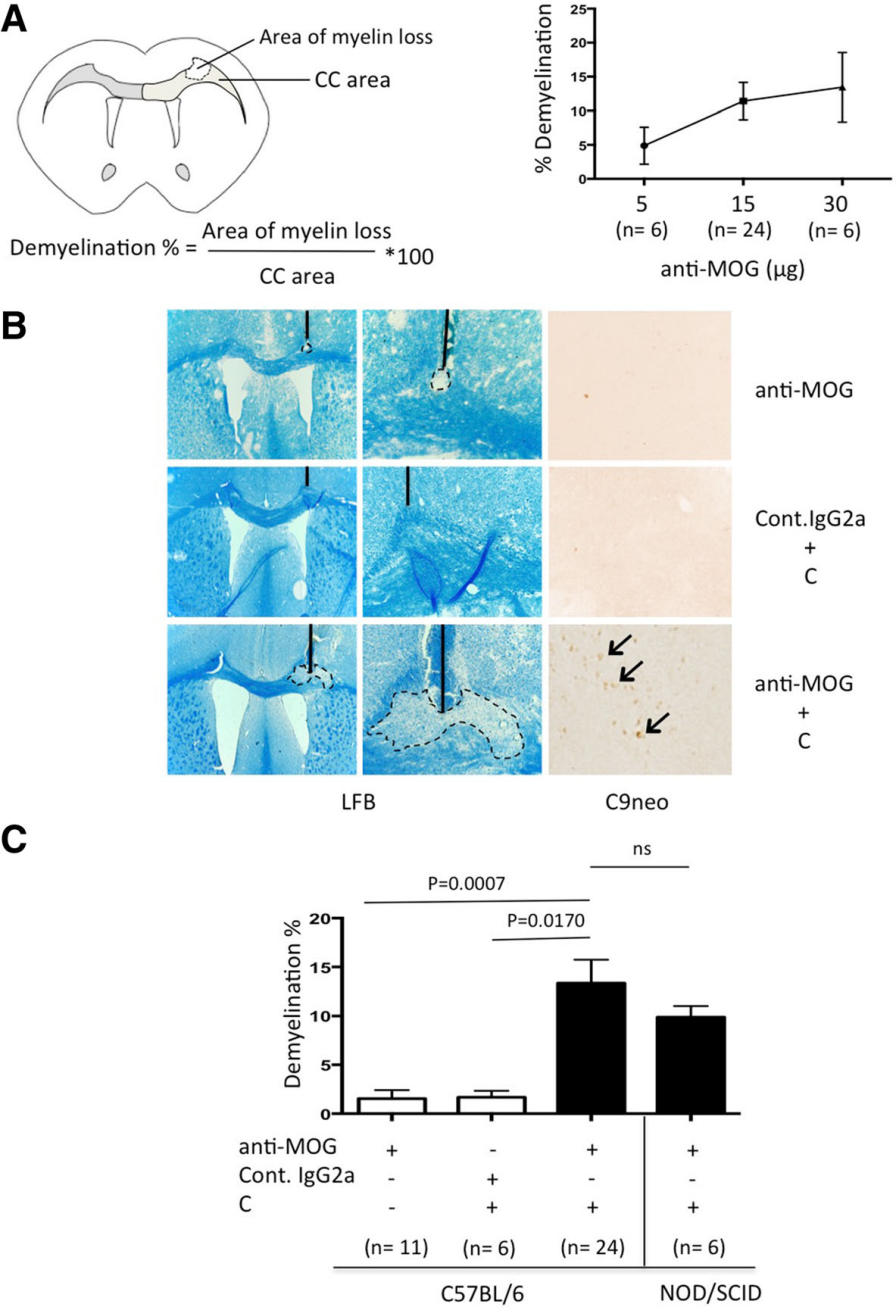
Anti-MOG + C-mediated demyelination. **a** The *left top panel* shows a schematic drawing of the experimental setup showing how demyelination was quantified. The degree of demyelination in percentage was measured as the loss of myelin (area of myelin loss) divided by area of corpus callosum (CC area) in that hemisphere of the brain. The *right top panel* shows a dose response for demyelination in CC of C57BL/6 mice receiving purified monoclonal anti-MOG antibody from hybridoma clone Z2, along with C. Data are shown as mean ± SEM, the total number of animals in each group is shown *underneath columns*, and this experiment was repeated only a single time with each dose. **b** Representative images of LFB and C9neo staining from C57BL/6 mice injected with anti-MOG, control IgG2a+C and anti-MOG + C. Magnification: LFB (×4 and ×20) and C9neo (×20). **c** Quantitation of the area of myelin loss in CC in C57BL/6 mice 2 days after injection of anti-MOG, control IgG2a+C, and anti-MOG + C as well as NOD-Scid/J immunodeficient mice injected with anti-MOG + C and pathology. Statistical significance was assessed using two-tailed Mann–Whitney *U* test. Results are shown as mean ± SEM, the total number of animals in each group is shown underneath columns. This experiment was repeated at least three times

To investigate whether a monoclonal MOG-specific antibody would be sufficient to induce MS-related pathology in the presence of C, we injected 5, 15, and 30 μg anti-MOG into CC and examined pathology 2 days later. Focal pathology corresponding to antibody and C-mediated damage was observed at the site of injection, in the CC. Significant demyelination was detectable at anti-MOG ≥15 μg, and this dose was used in all subsequent experiments (Fig. 1a). Demyelination was dependent on co-injection of C, and did not occur in mice that received anti-MOG alone, or control IgG2a with C (Fig. 1b, c). Deposition of activated C could be demonstrated at the same site as demyelination (Fig. 1b).

Some inflammation was seen, which was accounted for by the trauma of needle insertion.

**Fig. 2:**
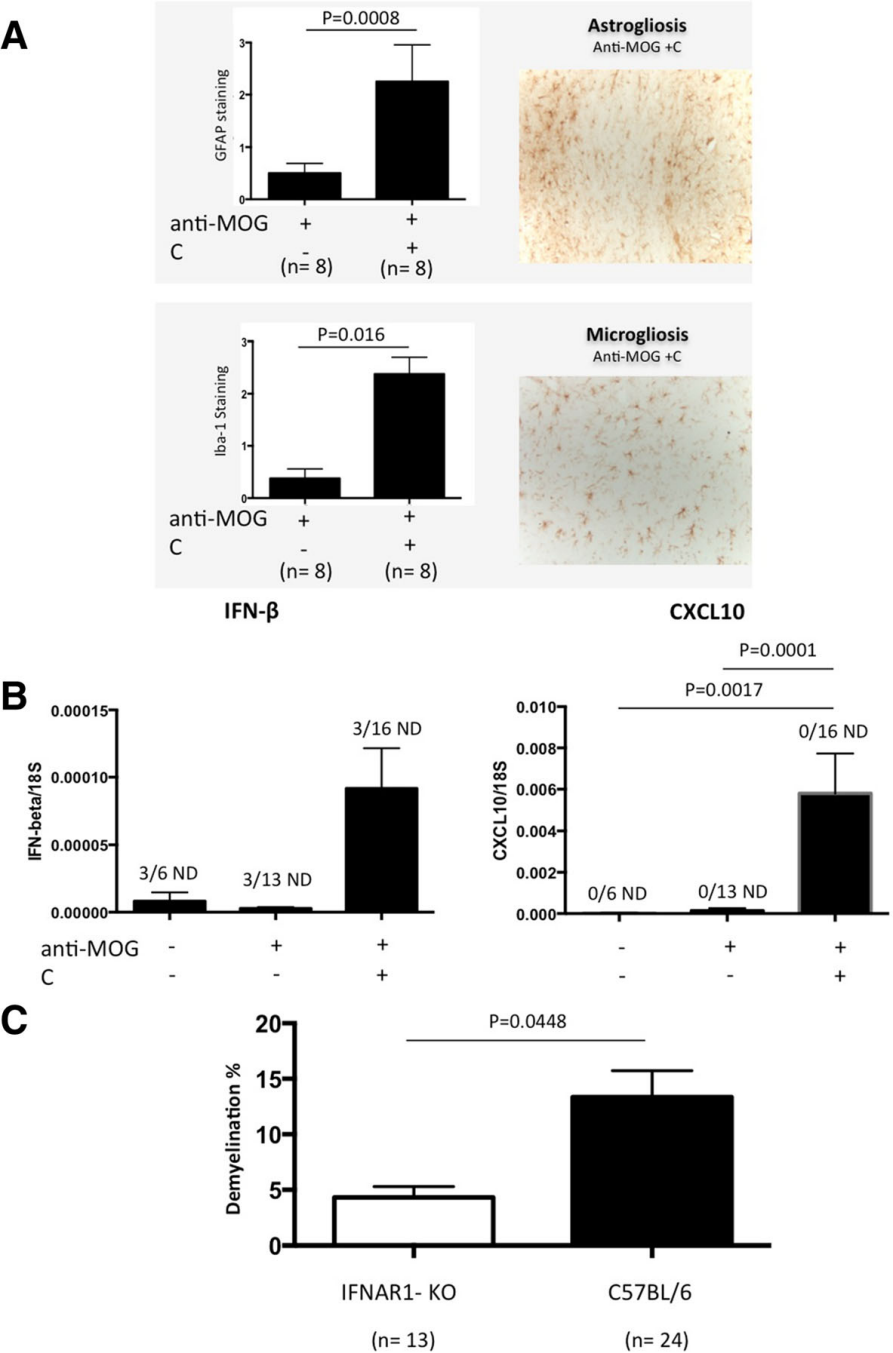
The influence of type I IFN signaling on antibody-mediated demyelination. **a** The *left panel* shows a *bar graph* with activation of astrocytes and microglia in and around demyelinated areas in animals injected with anti-MOG + C or anti-MOG alone. The *right panel* shows representative images of GFAP and Iba-1 staining from mice injected with anti-MOG + C. Results in the *bar graph* are shown as mean ± SEM of semi-quantitative scores (arbitrary units), the total number of animals in each group is shown *underneath columns*. Original magnification in the representative images is ×20. **b** *Bar graphs* showing IFN-β and CXCL10 gene expression in animals injected with anti-MOG + C, PBS, or anti-MOG alone. Data are shown as mean ± SEM, the total number of animals in each group is shown underneath columns. The data represent pooled results from at least three separate experiments. IFN-β gene expression was not detected (ND) in three samples from each group. **c** Anti-MOG + C was injected into CC of either C57BL/6 or IFNAR1-deficient mice, and pathology was assessed 2 days later. *Bar graph* shows percentage demyelination. Data were analyzed using two-tailed Mann–Whitney *U* test. Results are shown as mean + SEM, the total number of animals in each group is shown *underneath columns*. The data represent pooled results from three separate experiments

A few CD45-positive cells were located mainly within the needle track. We also observed activation of astrocytes (GFAP staining) and microglia (Iba1 staining) in and around demyelinated areas (Fig. 2a).

Demyelination was equivalent in NOD-Scid/J immunodeficient mice to which anti-MOG + C was injected into the CC (Fig. 1c). This shows that anti-MOG + C-mediated demyelination is independent of B and T cells.

### Induction of type I IFN and influence of type I IFN signaling on anti-MOG antibody-mediated demyelination

We used RT-qPCR to evaluate gene expression for IFN-beta (IFN-β), a type I IFN, and the IFN-induced chemokine CXCL10, in brain RNA isolates. We observed high expression of IFN-β mRNA compared to controls (Fig. 2b). Additionally, we observed a significant increase in gene expression for CXCL10. This points to a potential role for type I IFN signaling in antibody-mediated demyelination. We further investigated whether injection of anti-MOG + C led to pathology in IFNAR1-KO mice. Figure 2c shows that the lack of the IFNAR-1 receptor led to significantly reduced demyelination in CC, when compared to C57BL/6 mice.

### EAE and IFN-γ overcome dependency on type I IFN for anti-MOG + C induced demyelination

**Fig. 3:**
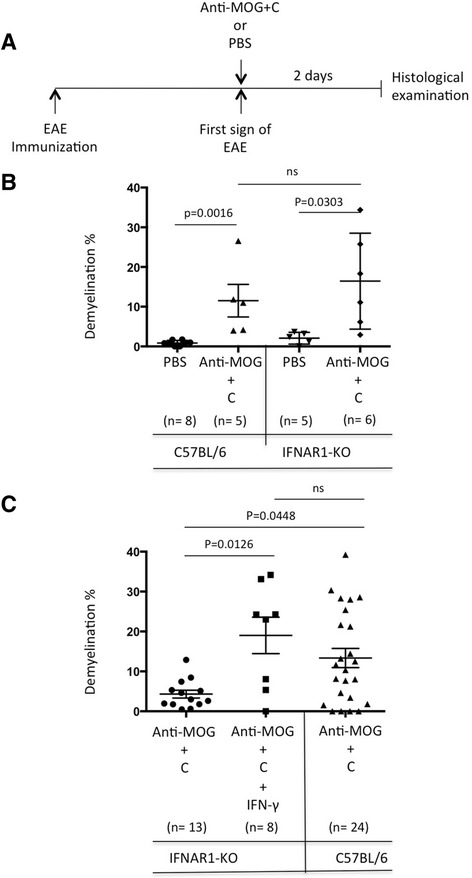
EAE or administration of IFN-γ overcomes dependence on type I IFN for anti-MOG + C-induced demyelination. **a** C57BL/6 and IFNAR1-KO mice were immunized with MOG_35–55_ as described in “Materials and methods.” At the onset of EAE, animals received either PBS or anti-MOG + C by stereotactic injection into CC. The mice were then monitored for symptoms daily and sacrificed after 2 days. **b** *Graphs* showing the quantification of demyelination. Data were analyzed using two-tailed Mann–Whitney *U* test. Results are shown as mean + SEM, the total number of animals in each group is shown *underneath columns*. The data represent pooled results from three separate experiments. **c** *Graph* shows quantitation of the area of myelin loss. Comparison of *first group * (IFNAR1-KO alone) with *middle *group (IFNAR1-KO + rIFNgamma (50 ng)) shows that induction of demyelination by the combination was equivalent to that in wild-type B6 mice (*third *group ). Statistical significance was assessed using two-tailed Mann–Whitney *U* test. Results are shown as mean + SEM, the total number of animals in each group is shown *underneath columns*. The data represent pooled results from at least three separate experiments

To test whether demyelination was similarly affected by type I IFN in mice with EAE, disease was induced by immunization with MOG peptide 35–55 and CFA. The incidence of EAE was almost two times higher in IFNAR1-KO mice compared to C57BL/6 mice (64.3 versus 33.9%). The time of onset of EAE was similar in C57BL/6 and IFNAR1-KO (13.4 ± 0.6 versus 14.8 ± 0.7 days). At onset of EAE, animals received anti-MOG + C or PBS by injection to CC (Fig. 3a). As expected, EAE was characterized by prominent mononuclear cell infiltration throughout the leptomeninges and white matter in the spinal cord (Additional file 1). EAE severity at day 2 was 2.2 ± 0.2 and 3.2 ± 0.6 for C57BL/6 mice injected with PBS and anti-MOG + C, respectively, and 2.3 ± 0.2 and 3.0 ± 0.4 for IFNAR1-KO injected with PBS and anti-MOG + C, respectively. Importantly there was no detectable pathology in the CC in mice with EAE unless induced by focal injection of anti-MOG and C (Fig. 3b). Callosal demyelination induced by anti-MOG + C in C57BL/6 mice with pre-established EAE was similar to that in mice without disease. Strikingly, the anti-MOG + C-induced callosal demyelination was not different in IFNAR1-KO with pre-established EAE compared to C57BL/6 mice.

The severity of EAE was not different between the groups treated with PBS or anti-MOG + C either in C57BL/6 or IFNAR1-KO, and the pattern of spinal cord infiltration in IFNAR1-KO and C57BL/6 mice was unaffected by intra-CC injection of anti-MOG + C or PBS (Additional file 1). To ask whether the equivalence of demyelination between WT and IFNAR1-KO mice with EAE could reflect inflammation-associated cytokines, we administered IFN-γ to IFNAR1-KO mice. Figure 3c shows that co-injection of IFN-γ into CC in IFNAR1-KO mice along with anti-MOG + C led to demyelination at levels equivalent to WT mice and so could compensate for the lack of type I IFN signaling.

## Discussion

We have shown that a MOG-specific antibody induced C-dependent demyelination in the CC of mice. Demyelination was similarly induced in NOD-Scid/J immunodeficient mice that lack T and B cells. We observed that the type I IFN receptor is required for anti-MOG + C-mediated demyelination. Remarkably, there was no effect of IFNAR1 deficiency on demyelination in CC in mice with pre-established EAE. Thus, induction of EAE overcame the dependence on type I IFN for anti-MOG + C-mediated demyelination in the CC. We could also overcome the effect of IFNAR1 deficiency by co-injection of IFN-γ. These findings show sufficiency of myelin-specific antibody with C for white matter demyelination and a role for IFN signaling in promoting this. These findings are novel, representing direct demonstration of pathogenicity of monoclonal anti-MOG antibody in otherwise unmanipulated mice.

It has previously been shown that co-injection of anti-MOG induced demyelination in an otherwise non-demyelinating uniphasic EAE model in Lewis rats [21]. Demyelination was observed in the perivascular cuffs and subpial bands of the white matter. Furthermore, high titer anti-MOG-containing guinea pig serum has been reported to induce demyelination when transferred into subarachnoid space of normal rats [22]. Demyelination that was observed in the spinal cord was presumed to reflect both the titer of antibody as well as serum complement. Our study has taken this approach further by using a monoclonal antibody of defined specificity in CC and examining the role of type I IFN.

In a previous study, we demonstrated type I IFN involvement in NMO-like pathology, showing reduced NMO-like pathology in mice lacking the receptor for type I IFN [24]. That finding was congruent with the clinical disease NMO, which is refractory to IFN-β treatment and may even be exacerbated by it [25, 30]. The involvement of type I IFN signaling in anti-MOG + C-induced demyelination may reflect a general role for IFN signaling. The mechanism whereby IFN signaling promotes complement-dependent antibody-mediated pathology, clearly of importance for inflammatory demyelinating diseases such as MS, remains to be established.

By contrast with NMO, IFN-β is used to treat MS and is effective against EAE. Over 50% of MS lesions have been shown to involve antibody and C deposition on demyelinating axons, so at first glance, it seems contradictory that IFN-β should have therapeutic benefit for MS while promoting antibody-mediated demyelination. However, therapeutic application of IFN-β in MS is via peripheral injection, and relapse prevention may depend on extra-CNS effects, as has been discussed [31]. Both type I and II IFNs are expressed in the CNS of MS patients [31, 32], so IFN-dependent pathology should not be impeded. Experimental models in which CNS-endogenous type I IFN has been shown to be protective are not noted for their dependence on antibody-mediated pathology [33] and indeed may not require antibodies or B cells at all [16]. Recently, it has been reported that treatment with IFN-β in MOG-IgG-positive patients was associated with increasing relapse rate [14]. It can be speculated that the lack of benefit of IFN-β in antibody-mediated diseases in CNS such as NMO may reflect the predominance of purely antibody-dependent pathology. Better definition of the relative roles of type I and II IFN in the CNS versus in the periphery will be required for fuller understanding of this complex interplay.

Stereotactic injection of a myelin-specific IgG antibody with C to a white matter tract has allowed analysis of focal demyelinating lesions that are independent of T or B cells. This experimental system identifies sufficiency of antibody and C for MS-like pathology and a role for IFN signaling in promoting this. These findings will need to be integrated to the much more complex inflammatory environment in MS in which other pathological mechanisms and many cell types also contribute.

## Additional file

Additional file 1: H&E and LFB staining of spinal cord sections from PBS- and anti-MOG + C-treated C57BL/6 and IFNAR1-KO mice with EAE. Shown is dorsal horn of the lumbar spinal cord taken 2 days post-intra-CC injection. There was no significant difference in EAE severity between IFNAR1-KO and C57BL/6 mice. EAE severity was 2.2 ± 0.2 and 3.2 ± 0.6 for C57BL/6 mice injected with PBS and anti-MOG + C, respectively, and 2.3 ± 0.2 and 3.0 ± 0.4 for IFNAR1-KO injected with PBS and anti-MOG + C, respectively. H&E and LFB magnification: ×4 and ×20. (JPEG 375 kb)

## Abbreviations

ADEM: Acute disseminated encephalomyelitis
AQP4: Aquaporin-4
C: Complement
CC: Corpus callosum
CNS: Central nervous system
CSF: Cerebrospinal fluid
EAE: Experimental autoimmune encephalomyelitis
H&E: Hematoxylin and eosin
IFN: Interferon
IFNAR1-KO: Type I IFN receptor deficient mice
IFN-β: IFN-beta
IFN-γ: IFN-gamma
IgG: Immunoglobulin G
LFB: Luxol fast blue
MOG: Myelin oligodendrocyte glycoprotein
MS: Multiple sclerosis
NMO: Neuromyelitis optica
NMOSD: Neuromyelitis optica spectrum disorder
PBS: Phosphate-buffered saline
SEM: Standard error of mean
