# Supplementary figures and images for: Influence of type I IFN signaling on anti-MOG antibody-mediated demyelination
Source: J Neuroinflammation. 2017 Jun 24;14:127. doi: 10.1186/s12974-017-0899-1 (PMC5483301; doi:10.1186/s12974-017-0899-1)

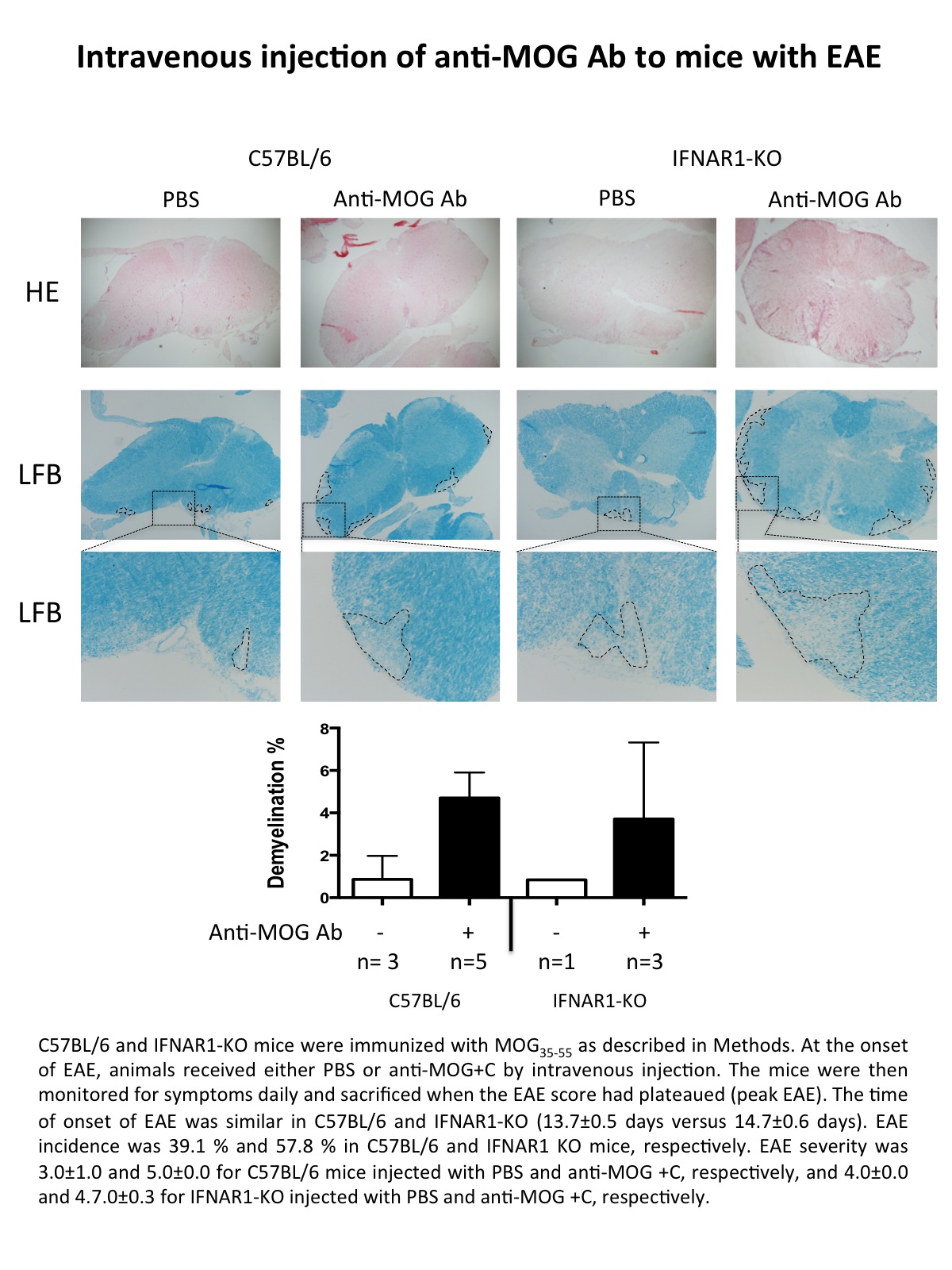

Supplement: Additional file 1: — H&E and LFB staining of spinal cord sections from PBS- and anti-MOG + C-treated C57BL/6 and IFNAR1-KO mice with EAE. Shown is dorsal horn of the lumbar spinal cord taken 2 days post-intra-CC injection. There was no significant difference in EAE severity between IFNAR1-KO and C57BL/6 mice. EAE severity was 2.2 ± 0.2 and 3.2 ± 0.6 for C57BL/6 mice injected with PBS and anti-MOG + C, respectively, and 2.3 ± 0.2 and 3.0 ± 0.4 for IFNAR1-KO injected with PBS and anti-MOG + C, respectively. H&E and LFB magnification: ×4 and ×20. (JPEG 375 kb) [file 12974_2017_899_MOESM1_ESM.jpeg]
